# Standardized Patients to Assess Resident Interpersonal Communication Skills and Professional Values Milestones

**DOI:** 10.5811/westjem.2018.8.37204

**Published:** 2018-10-18

**Authors:** Samreen Vora, Matt Lineberry, Valerie Ann Dobiesz

**Affiliations:** * Children’s Minnesota, Department of Simulation, Minneapolis, Minnesota; † University of Kansas Medical Center and Health System, Zamierowski Institute for Experiential Learning, Kansas City, Kansas; ‡ Brigham & Women’s Hospital, STRATUS Center for Medical Simulation, Department of Emergency Medicine, Boston, Harvard Medical School, Massachusetts

## Abstract

It has been a challenge to assess communication and professional values Milestones in emergency medicine (EM) residents using standardized methods, as mandated by the Accreditation Council for Graduate Medical Education (ACGME). This paper outlines an innovative method of assessing these Milestones using an established instructional method. EM faculty mapped the communication and professional values Milestones to an existing communication and interpersonal skills scale. We identified six communication-focused scenarios: death notification; informed consent; medical non-compliance; medical error; treatment refusal; and advanced directives. In a pilot, 18 EM residents completed these six standardized patient (SP) encounters. Our experience suggests SP encounters can support standardized direct observation of residents’ achievement of ACGME Milestones. Further effort can be made to create a tailored, behaviorally-anchored tool that uses the Milestones as the conceptual framework.

## INTRODUCTION

Although effective communication with patients is an integral part of the role of all physicians and has gained the spotlight over the last decade, there is no established standard on how it should be taught and assessed during traditional medical training.^1^–^6^ The urgency to address this gap is evident, as the literature indicates that deficiencies in communication skills can lead to higher malpractice rates, patient dissatisfaction, and adverse patient outcomes.^7^–^9^ The Accreditation Council for Graduate Medical Education (ACGME) endorsed “Interpersonal and Communication Skills” and “Professionalism” as two of the six core competencies. These competencies, and newly mandated ACGME Milestones, are challenging to assess in the clinical setting due to varying faculty frames of reference and the influence of factors external to resident performance.^10^ Faculty may use themselves, other doctors, or patient outcomes as frames of reference when assessing residents. In addition, faculty report that they often use “gut feeling” or “gestalt” to translate their observations to numerical assessment scores.^10^

Standardized patient (SP) encounters with validated tools are an established method of assessing learners^11^ and may offer a more consistent way to assess residents. The literature supports a correlation between patient surveys and SP-based assessments of learners,^12^ as well as the use of SP feedback for training and assessment of residents.^13^–^16^ Using this established method can offer a more reliable assessment of these residency Milestones. This project aimed to pilot an innovative SP-based model to assess the interpersonal communication skills and professionalism Milestones of emergency medicine (EM) residents.

## METHODS

In 2005, the University of Illinois-College of Medicine at Chicago (UIC-COM) Clinical Performance Center (CPC) developed an institution-based competency tool to provide resident performance data to program directors (PD). The Communication and Interpersonal Skills Objective Structured Clinical Exam (CIS-OSCE) was administered and analyzed across specialties including internal medicine, family medicine, surgery, pediatrics, neurology, and obstetrics-gynecology.^17^ Later, a many-faceted Rasch measurement model was used to further analyze each item on the scale and the results of this analysis were used to create an improved communication rating scale.^18^ We used the new Revised Communication and Interpersonal Skills (RUCIS) scale, a four-category behaviorally anchored rating scale (Table).

Using a mapping method, four EM academic residency faculty integrated the Milestones into this existing RUCIS scale. Already being familiar with the Interpersonal and Communication Skills and Professionalism Milestones, the faculty members were given a chance to review the RUCIS Scale (with no modification to the anchors) and the details of the six communication tasks. They were then asked to individually map each of the behaviorally-anchored ratings on the RUCIS scale to a specific Milestone and level. Then the mapping was reviewed as a group and consensus was reached through iterative discussion until consensus was reached among all faculty (Table 1). Eleven of the 13 RUCIS items were successfully mapped to specific levels of the two EM Milestones (Milestone 20 PROF1 and Milestone 22 ICS1). In the 11 items, Levels 1 through 4 of both Milestones were represented, with Milestone 20 measured five times and Milestone 22 measured six times.

In parallel to the Milestone mapping, the communication challenges were identified and developed. The six communication tasks were identified based on the previously developed patient-centered communication competency assessment implemented in the CPC in 2003. The tasks were originally identified based on the communication literature and their salience to clinical practice. As noted by the authors, “they were designed to allow residents to demonstrate their skills across a range of patient ages, genders, and problems”.^9^ For our Milestone assessment initiative, the tasks used were the following: giving bad news; obtaining informed consent; patient education (addressing medication non-compliance); medical error; treatment refusal; and advanced directives. The cases for each of these tasks were either adapted to the EM setting from previous cases used by other specialties or were newly created and validated by EM faculty through iterative review. Each case was designed to present a communication task with an underlying communication challenge. For example, the communication task in the “giving bad news” case was for the resident to appropriately deliver a death notification and the communication challenge was for the resident to address the need for an autopsy of the deceased.

Each SP encounter was 10 minutes, with the SP completing the RUCIS scale immediately following the encounter. This was followed by 10 minutes for SP-to-resident debriefing. Professional actors were trained by an EM faculty member and an experienced SP trainer on each of the six cases. During the rigorous training, the actors reviewed and practiced the standardized scripting of each case and were tested on their accuracy and standardized portrayal of the patient. The SPs completed rater training for the RUCIS scale, which entailed discussing examples of each item and score with the trainers and watching video examples. The SPs were also trained in techniques of providing feedback to the residents according to the CPC protocol. A convenience subset of encounters were observed by an EM faculty member. At each session six residents rotated through the encounters and concluded the half day with an individual survey of their experience and a group debriefing.

Piloting consisted of 18 residents representing all levels of EM residents or combined EM/Internal Medicine residents in the University of Illinois at Chicago Program (seven postgraduate year [PGY]-1 residents, six PGY-2, and five PGY-3). The six cases were new to all resident participants. Residents were assigned a simulation time slot during which they were excused from clinical duties. At the end of the academic year, the data were forwarded to the EM PD for use during the annual evaluation process for individual residents overseen by the Clinical Competency Committee (CCC) meeting. The scores were averaged across the six cases using the mapped Milestone level, and the resident’s level on Milestone 20 and Milestone 22 were reported separately.

This study was included under the Clinical Performance Center Institutional Review Board (or human subjects committee) approval.

## RESULTS

As this was intended as a pilot of an innovative Milestone assessment method, the sample size was small and collected data was limited. An individualized score report was provided to the CCC for each resident that included the Milestone score for each of the two Milestones. See Table 2 as an example. The score report was included for faculty to review as part of the resident’s file; but as this was a pilot, it was not incorporated in any specific numerical way into the resident’s overall Milestone score.

Additionally, in the individual survey 94% of residents agreed that verbal feedback from the SP was helpful and 100% of residents felt the cases allowed them to demonstrate their communication and professionalism skills. In the faculty debriefing, residents uniformly agreed the SP encounter and feedback would improve the quality of care for future patients.

## DISCUSSION

In this program, essential communication skills were assessed and EM residents received feedback from the SP as well as an EM faculty member in a simulated setting. This paper demonstrates the utilization of an established OSCE method for Milestone assessment that could provide useful, quantitative performance data to a residency CCC. Although the CCC did not use this pilot data in a structured way, there is potential for standardized incorporation of these scores in the future. Using a larger sample size, it would be important to look at the correlation of individual resident OSCE scores with other assessments in their file.^19^ Other possibilities would be to correlate resident scores to level of experience and comfort. Although in this pilot variability in resident scores across experience level was noted, the sample size was too small to report any statistically significant correlation data. Also, SPs were able to offer targeted feedback to individual learners and a difficult-to-obtain patient perspective. This method of assessment is reported in the literature,^13^,^14^ but further study is needed to assess resident communication skills Milestone improvement after SP debriefing.

In this pilot, a previously created tool was mapped to the Milestones. Since patient-centered care was the conceptual framework for the RUCIS scale and not the ACGME EM Milestones, it is necessarily limited in directly assessing ACGME EM Milestones. Further work is needed to create a new, targeted assessment tool that can be used in conjunction with the established OSCE methodology to specifically assess interpersonal communication and professional values Milestones. Using the Milestones as a conceptual framework, a behaviorally anchored tool could be created, similar to the CIS-OSCE, to assess specific behaviors as outlined by the ACGME Milestones. This type of tool could provide consistent, reliable, quantitative data to residency PDs and enhance the instruction and assessment of residents throughout their training with the ultimate goal of improving these skills in patient interactions.

## LIMITATIONS

As this was a pilot study, there was an anticipated limitation in sufficient data collection to perform definitive quantitative analyses. Residents knew they were scheduled for communication OSCE encounters and may have focused on demonstrating strong communication. Thus, like many standardized assessments these can be best thought of as “maximal performance” assessments, which may not reflect learners’ typical performance or “worst-case” performance.^20^ Also, as with many OSCEs, it is possible that residents who were scheduled earlier discussed some case content with later-scheduled residents although the scores of later residents did not reflect this to be the case.

Of note, when the Milestones were mapped to the RUCIS scale, level 5 of both Milestones was not represented in the current tool, although it is notable that residents are not expected to reach level 5 during residency training. This may speak to the need to develop specific assessment methods to measure higher level Milestones with OSCE assessments used for early level Milestones. In addition, the scores provided to the CCC reported a number as a continuous variable as opposed to an ordinal variable as required by the Milestone scale, which may have limited their usefulness. In the future, qualitative feedback from the PD or the CCC on the value of the mapped OSCE score could inform score report structure. This program was piloted at one institution, which would limit its generalizability. Due to these limitations, it would be worthwhile to explore creating a new assessment tool with the EM Milestones as the underlying conceptual framework.

## CONCLUSION

As competency-based medical education has come to the forefront, there is a need for reliable and valid methods of assessing communication and professionalism skills. This pilot supports the potential use of an established method to conduct a more rigorous assessment of interpersonal communication skills and professional values Milestones of EM residents. Future studies may also compare SP assessment to standard simulation assessment of these skills to further expand the Milestone assessment toolbox.

## Figures and Tables

**Table 1:** Snapshot of RUCIS, a behaviorally-anchored rating scale mapped to milestones. (For quick reference to Milestone description and anchors, please use this link: https://www.acgme.org/Portals/0/PDFs/Milestones/EmergencyMedicineMilestones.pdf).

		Milestone: level
3. Listening to my story	( ) You rarely gave me any opportunity to tell my story and/or frequently interrupted me while I was talking, not allowing me to finish what I was saying. Sometimes I felt you were not paying attention (for example, you asked for information that I already provided).	22:0
	( ) You let me tell my story without interruption, or only interrupted appropriately and respectfully. You seemed to pay attention to my story and responded to what I said appropriately.	22:1
	( ) You allowed me to tell my story without inappropriate interruption, responded appropriately to what I said, and asked thoughtful questions to encourage me to tell more of my story.	22:2
	( ) You were an exceptional listener. You encouraged me to tell my story and checked your understanding by restating important points.	22:3
4. Honest communication	( ) You did not seem truthful and frank. I felt that there might be something that you were trying to hide from me.	20:0
	( ) You did not seem to hide any critical information from me.	20:0
	( ) You explained the facts of the situation without trivializing negative information or possibilities (e.g., side effects, complications, failure rates).	20:2
	( ) You were exceptionally frank and honest. You fully explained the positive and negative aspects of my condition. You openly acknowledged your own lack of knowledge or uncertainty, and things you would have to consult with others. When appropriate, you also suggested I seek a second opinion.	20:4
	( ) Not applicable. There was no information for the clinician to provide.	N/A
5. Interest in me as a person	( ) You never showed interest in me as a person. You only focused on the disease or medical issue.	20:0
	( ) In addition to talking about my medical issue, you spent some time getting to know me as a person.	20:2
	( ) You spent some time exploring how my medical issue affects my personal or social life.	20:3
	( ) You were exceptionally interested in me as a person. You not only explored how my medical problem affects my personal and social life, but also showed your willingness to help me address those challenges.	20:4

*RUCIS*, Revised Communication and Interpersonal Skills scale.

**Table 2:** Sample resident score report.

Resident X Score Report: (Average score across 6 cases based on Milestone levels 1–5)
Milestone 20 (Professional Values) – 2.23
Milestone 22 (Patient-centered Communication) – 2.15
CIS score: 74%

## References

[1] Wolff M, Macias CG, Garcia W (2014). Patient safety training in pediatric emergency medicine: a national survey of program directors. Acad Emerg Med.

[2] Gaiser RR (2009). *T* he teaching of professionalism during residency: why it is failing and a suggestion to improve its success. Anesth Analg.

[3] Morgan ER, Winter RJ (1996). Teaching communication skills: an essential part of residency training. Arch Pediatr Adolesc Med.

[4] Klein EJ, Jackson JC, Kratz L (2003). Teaching professionalism to residents. Acad Med.

[5] Lee AG, Beaver HA, Boldt HC (2007). Teaching and assessing professionalism in ophthalmology residency training programs. Surv Ophthalmol.

[6] Hochberg MS, Kalet A, Zabar S (2010). Can professionalism be taught? Encouraging evidence. Am J Surg.

[7] Kohn LT, Corrigan JM, Donaldson MS (2000). To err is human: building a safer health system. A report of the Committee on Quality of Health Care in America, Institute of Medicine.

[8] Commission J (2005). Communication: a critical component in delivering quality care. Joint Commission: The Source.

[9] Cox LM, Logio LS (2011). Patient safety stories: a project utilizing narratives in resident training. Acad Med.

[10] Kogan JR, Conforti L, Bernabeo E (2011). Opening the black box of clinical skills assessment via observation: a conceptual model. Med Educ.

[11] Downing SM, Yudkowsky R (2009). Assessment in health professions education.

[12] Dine CJ, Ruffolo S, Lapin J (2014). Feasibility and validation of real-time patient evaluations of internal medicine interns’ communication and professionalism skills. J Grad Med Educ.

[13] Leeper-Majors K, Veale JR, Westbrook (2003). The effect of standardized patient feedback in teaching surgical residents informed consent: results of a pilot study. Curr Surg.

[14] Barry CT, Avissar U, Asebrook MI (2010). Use of a standardized patient exercise to assess core competencies during fellowship training. J Grad Med Educ.

[15] Doyle Howley L, Martindale J (2004). The efficacy of standardized patient feedback in clinical teaching: a mixed methods analysis. Med Educ Online.

[16] Turan S, Üner S, Elçin M (2009). The Impact of standardized patients’ feedback on the students’ motivational levels. Procedia Soc Behav Sci.

[17] Yudkowsky R, Downing SM, Sandlow LJ (2006). Developing an institution-based assessment of resident communication and interpersonal skills. Acad Med.

[18] Iramaneerat C, Myford CM, Yudkowsky R (2009). Evaluating the effectiveness of rating instruments for a communication skills assessment of medical residents. Adv Health Sci Educ Theory Pract.

[19] Duffy FD, Gordon GH, Whelan G (2004). Assessing competence in communication and interpersonal skills: the Kalamazoo II Report. Acad Med.

[20] DuBois CL, Sackett PR, Zedeck S (1993). Further exploration of typical and maximum performance criteria: definitional issues, prediction, and white-black differences. J Appl Psychol.

